# *SMARCB1*‐deficient basal cell carcinoma of the prostate controlled using radiation therapy

**DOI:** 10.1002/iju5.12598

**Published:** 2023-06-14

**Authors:** Shunta Makabe, Tomoyuki Koguchi, Kanako Matsuoka, Seiji Hoshi, Junya Hata, Yuichi Sato, Hidenori Akaihata, Masao Kataoka, Motohide Uemura, Yoshiyuki Kojima

**Affiliations:** ^1^ Department of Urology Fukushima Medical University School of Medicine Fukushima Japan

**Keywords:** basal cell carcinoma, *GLI1*, prostate adenocarcinoma, radiotherapy, *SMARCB1*

## Abstract

**Introduction:**

Basal cell carcinoma of the prostate is rare, with no established treatment for its recurrence or metastasis. We report a case involving basal cell carcinoma of the prostate controlled using radiotherapy.

**Case presentation:**

A 57‐year‐old man complained of perineal pain. Although his prostate‐specific antigen was 0.657 ng/mL, a digital rectal examination revealed his prostate was stone hard. Prostate needle biopsy showed basal cell carcinoma of the prostate. The patient then underwent radical prostatectomy. Local recurrence and sacral bone metastasis appeared 2 months after surgery. OncoGuide™ NCC Oncopanel System showed deletion of *SMARCB1*; however no recommended treatment was identified. Thus, we decided to perform radiotherapy, which reduced all lesions.

**Conclusion:**

Basal cell carcinoma of the prostate may have a poor prognosis with recurrence or metastasis, hence evaluation of prognostic factors is important. In this case, the genomic profiling test suggested that *SMARCB1* deletion may be a prognostic factor associated with disease progression.

**Keynote message**

When basal cell carcinoma (BCC) of the prostate recurs or metastases, the prognosis is poor, and no treatment is available. Therefore, the search for prognostic factors and treatments is important. We describe a case of BCC of the prostate, in which radiotherapy was used to control the disease and a genomic profiling test was used to investigate treatment and prognostic factors.

**Abbreviations & Acronyms**
BCC: basal cell carcinoma
CT: computed tomography
ISUP: International Society of Urological Pathology
MRI: magnetic resonance imaging
PSA: prostate‐specific antigen
RT‐PCR: real‐time polymerase chain reaction

## Case presentation

**Fig. 1:**
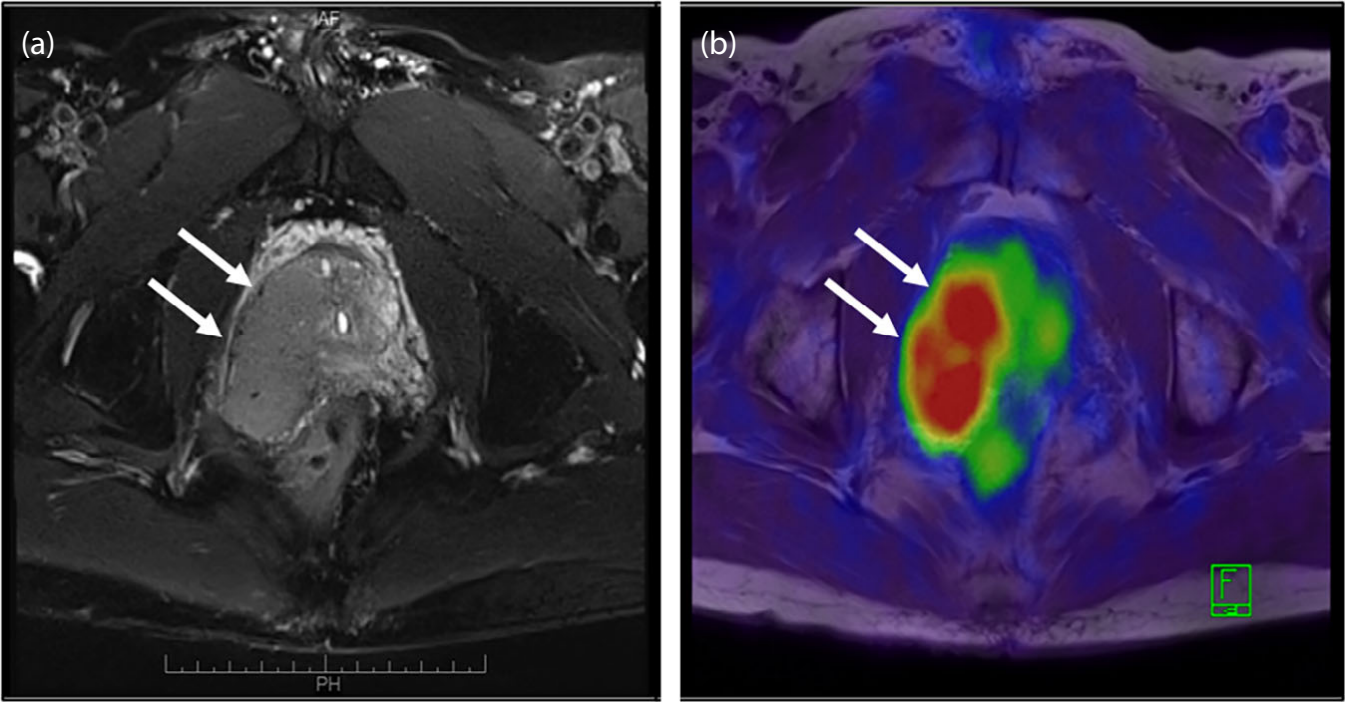
Preoperative fluorodeoxyglucose‐positron emission tomography/MRI. (a) T2‐weighted low‐signal lesion in the right lobe. (b) FDG accumulation with SUV max 7.1.

A 57‐year‐old man presented to our hospital complaining of bowel movements accompanied by perineal pain. Although his PSA level was 0.657 ng/mL, a digital rectal examination showed that the prostate was as stone hard. T2‐weighted low‐signal and diffusion‐weighted high‐signal MRI showed a mass in the right lobe of the prostate. Prostate needle biopsy suspected BCC. Immunohistochemical staining was negative for PSA, chromogranin A, synaptophysin, and uroplakin, but positive for p63, bcl‐2, and Ki67 (>20%). CT and fluorodeoxyglucose‐positron emission tomography/MRI suspected right seminal vesicle invasion; however, no metastasis was observed (Fig. 1). Therefore, the patient was diagnosed with BCC of the prostate (cT3bN0M0) and was scheduled to undergo radical prostatectomy. The final pathological diagnosis was BCC of the prostate, with extraprostatic extension, positive resection margins, lymphatic and venous invasion, and perineural invasion. Right internal iliac lymph node metastasis was pathologically observed. Immunohistochemical staining was negative for PSA, but positive for p63, bcl‐2, and Ki67 (>20%).

**Fig. 2:**
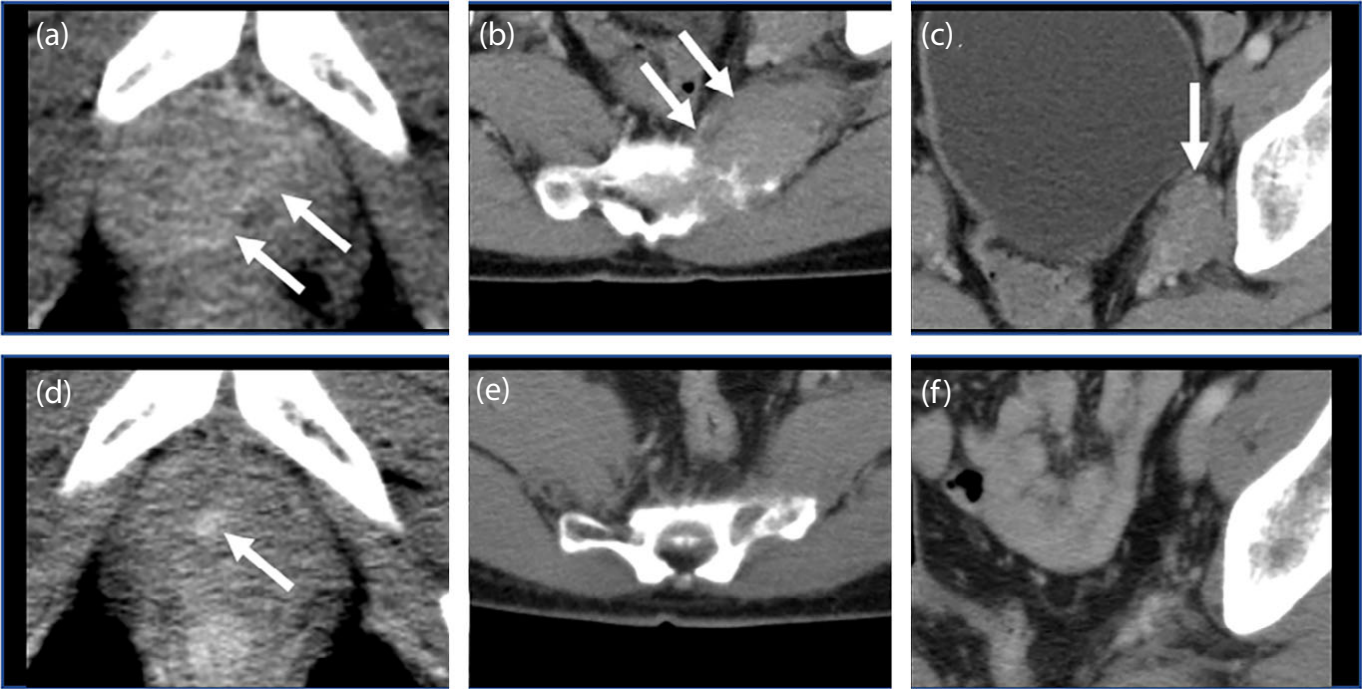
Pelvic CT before (a–c) and after (d–f) radiation therapy. Local recurrence on the dorsal pubis (a, d). Bone metastasis on the left side of the sacrum. (b, e). Left closed lymph node metastasis (c, f).

Two months after surgery, CT showed local recurrence on the dorsal pubis, sacral bone metastasis, and left obturator nerve lymph node metastasis (Fig. 2). Since no effective treatment for BCC of the prostate has been reported, we performed the OncoGuide™ NCC Oncopanel System, a genomic profiling test by Japan's national cancer center, to search for targets of treatment. Accordingly, our findings revealed *SMARCB1* (SWI/SNF related, matrix associated, actin dependent regulator of chromatin, subfamily b, member 1) deletion, for which no recommended treatment has been established. Therefore, we decided to use radiotherapy (66 Gy to the entire pelvis) to treat local recurrence and metastatic lesions, which reduced the size of these lesions (Fig. 2). During the 8 months after radiotherapy, the patient showed no signs of disease progression.

**Fig. 3:**
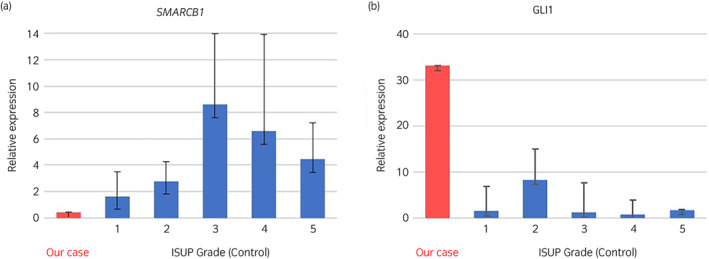
Real‐time polymerase chain reaction comparing mRNA expression of *SMARCB1* (a) and *GLI1* (b) in this case and prostate adenocarcinoma (5 cases each from ISUP grade group 1–5 in prostate adenocarcinoma specimens).

**Fig. 4:**
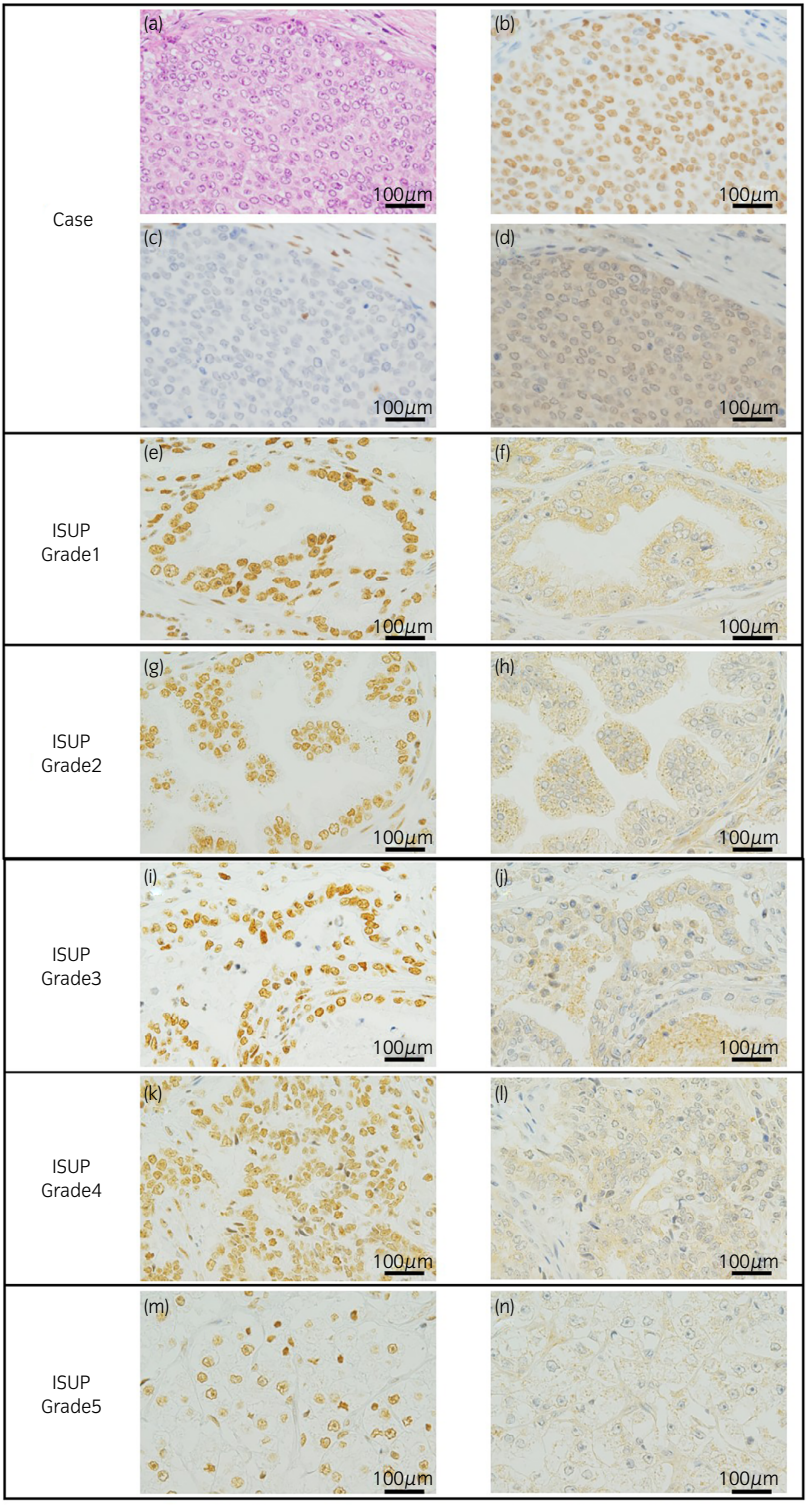
Pathology and immunohistochemical staining in prostatectomy specimens (5 cases each from ISUP grade group 1–5). (a) Hematoxylin and eosin staining (magnification: ×400); (b) immunohistochemistry for p63 (magnification: ×400); (c, e, g, i, k, m) immunohistochemistry for *SMARCB1* (magnification: ×400); (d, f, h, j, l, n) immunohistochemistry for GLI1 (magnification: ×400).

Because a genomic profiling test revealed *SMARCB1* deletion, we examined the protein and mRNA levels of *SMARCB1* and *GLI1* (GLI family zinc finger 1; a gene repressed by *SMARCB1*). RT‐PCR was used to analyze samples from resected tissues during a prostatectomy. In this case, the tissues had lower *SMARCB1* and higher *GLI1* compared with prostate adenocarcinoma tissues in ISUP grade groups 1–5 (Fig. 3). Furthermore, SMARCB1 and GLI1 were evaluated by immunohistochemical staining (Fig. 4). In this case, SMARCB1 was negative and GLI1 was strongly positive when compared to prostate adenocarcinoma tissues in ISUP grade groups 1–5.

## Discussion

BCC of the prostate is an exceedingly rare type of tumor, accounting for approximately 0.01% of all prostate cancer cases.^1^ This rare cancer has been described in only approximately 140 cases in the online literature search database PubMed.^2^ While these lesions have an indolent course, others have shown a progressive course, including recurrence and metastasis. Despite previous reports showing a good prognosis for BCC of the prostate,^3^ recent reports have shown a poor prognosis for the disease. In addition, there is no established treatment for recurrence or metastasis.^4^ Therefore, we believe it necessary to discuss factors influencing disease progression and treatments.

First, we discussed prognostic factors for BCC of the prostate. BCC of the prostate exhibits a variety of histological patterns, such as adenoid cystic carcinoma pattern, basal cell hyperplasia pattern, and large solid nests with necrosis. Immunohistochemical staining has shown high positivity rates for basal cell markers bcl2 and p63. Ki67 is also useful, with half of the cases showing a positive rate of >20%. Based on histopathological studies, poor prognostic factors reported include large solid nests, necrosis, Ki67 positivity of >20%, and negative for p63.^3^ In our case, we found large solid nests with necrosis and a Ki67 positivity rate of 33%. These factors could have promoted disease progression.

Thereafter, we discussed the genetic mutation observed in this case. The OncoGuide™ NCC Oncopanel System showed *SMARCB1* deletion. *SMARCB1*, is one of the components of the SWI/SNF complex, and is involved in various transcriptional regulatory and transduction pathways.^5^, ^6^ Moreover, reports have shown that loss of *SMARCB1* affects the development and progression of tumors, such as malignant rhabdoid tumors, epithelioid sarcoma, sinonasal basaloid carcinoma, etc.^7^ One of the transduction pathways is the hedgehog pathway, which is involved in the differentiation and proliferation of normal cells. *SMARCB1* directly represses *GLI1*, a transcription factor that exists downstream of the hedgehog pathway. *GLI1* is involved in normal cell proliferation and patterning and the growth of *SMARCB1*‐deficient tumor cells. *GLI1* has also been reported to affect cancer progression via the hedgehog pathway.^8^ In this case, *SMARCB1* expression was lower and *GLI1* expression was higher in immunohistochemical staining and RT‐PCR compared with prostate adenocarcinoma tissues. Therefore, this suggests that *GLI1* overexpression caused by *SMARCB1* deficiency affected disease progression. This is the first report indicating that *SMARCB1* deficiency may be a poor prognostic factor in prostate cancer.

We then discussed the treatment for BCC of the prostate. No standard treatment has been reported for BCC of the prostate. For advanced cases, multidisciplinary treatment, such as surgery, radiotherapy, and chemotherapy are generally used; however, there have been few reports on the secondary treatment after recurrence or metastasis. There are no reports on the effectiveness of radiotherapy as a treatment for BCC of the prostate, and more research is needed. Because the OncoGuide™ NCC Oncopanel System did not recommend any therapy for *SMARCB1* deletion in this case, it is unclear whether this mutation will result in targeted therapy. However, clinical trials of vismodegib, a hedgehog pathway inhibitor for BCC of the skin,^9^ and tazemetostat, an EHZ2 inhibitor for *SMARCB1*‐deficient tumors, are presently underway,^10^, ^11^ and targeted therapy may also be applicable in the prostate.

## Conclusion

A genomic profiling test, in this case, suggested that the mutation associated with disease progression, *SMARCB1* deficiency, could be a useful poor prognostic factor. Future investigation into the possibility of targeted therapy and the efficacy of radiotherapy in treating this cancer is required.

## Author contributions

Shunta Makabe: Conceptualization; data curation; formal analysis; funding acquisition; investigation; project administration; writing – original draft; writing – review and editing. Tomoyuki Koguchi: Data curation; writing – review and editing. Kanako Matsuoka: Data curation. Seiji Hoshi: Data curation. Junya Hata: Data curation. Yuichi Sato: Data curation. Hidenori Akaihata: Data curation. Masao Kataoka: Data curation. Motohide Uemura: Supervision. Yoshiyuki Kojima: Conceptualization; supervision; writing – review and editing.

## Conflict of interest

The authors declare no conflict of interest.

## Approval of the research protocol by an Institutional Reviewer Board

Not applicable.

## Informed consent

Not applicable.

## Registry and the Registration No. of the study/trial

Not applicable.
